# COVID-19 Pandemic and Patients with Rare Inherited Metabolic Disorders and Rare Autoinflammatory Diseases—Organizational Challenges from the Point of View of Healthcare Providers

**DOI:** 10.3390/jcm10214862

**Published:** 2021-10-22

**Authors:** Ewa Tobór-Świętek, Jolanta Sykut-Cegielska, Mirosław Bik-Multanowski, Mieczysław Walczak, Dariusz Rokicki, Łukasz Kałużny, Joanna Wierzba, Małgorzata Pac, Karina Jahnz-Różyk, Ewa Więsik-Szewczyk, Beata Kieć-Wilk

**Affiliations:** 1 Department of Metabolic Diseases, Jagiellonian University Medical College, 31-008 Cracow, Poland; ewa.tobor@doctoral.uj.edu.pl; 2 Department of Metabolic Diseases, University Hospital, Jakubowskiego 2 Street, 30-688 Cracow, Poland; 3 Department of Inborn Errors of Metabolism and Paediatrics, Institute of Mother and Child, Kasprzaka 17a Street, 01-211 Warsaw, Poland; jolanta.cegielska@imid.med.pl; 4 Department of Medical Genetics, Medical College, Jagiellonian University, Wielicka 265 Street, 30-633 Cracow, Poland; miroslaw.bik-multanowski@uj.edu.pl; 5 Department of Pediatrics, Endocrinology, Diabetology, Metabolic Diseases and Cardiology of the Developmental Age, Pomeranian Medical University, Unii Lubelskiej 1 Street, 71-242 Szczecin, Poland; ghmwal@pum.edu.pl; 6 Department of Paediatrics, Nutrition and Metabolic Diseases, Institute “Children’s Memorial Health Institute”, al. Dzieci Polskich 20, 04-730 Warsaw, Poland; d.rokicki@ipczd.pl; 7 Department of Pediatric Gastroenterology and Metabolic Diseases, Poznan University of Medical Sciences, Szpitalna 27/33 Street, 60-572 Poznan, Poland; lukasz@jerozolima.poznan.pl; 8 Department of Paediatrics, Hematology and Oncology Medical University of Gdansk, Debniki 7, 80-752 Gdansk, Poland; jolanta.wierzba@gumed.edu.pl; 9 Department of Immunology, Institute “Children’s Memorial Health Institute”, al. Dzieci Polskich 20, 04-730 Warsaw, Poland; m.pac@ipczd.pl; 10 Department of Internal Diseases, Pneumology, Allergology and Clinical Immunology, Military Medical Institute, Szaserów 128 Street, 04-141 Warsaw, Poland; kjrozyk@wim.mil.pl (K.J.-R.); ewa.w.szewczyk@gmail.com (E.W.-S.); 11 Unit of Rare Metabolic Diseases, Department of Metabolic Diseases Jagiellonian University, Medical College, University Hospital, Jakubowskiego 2 Street, 30-688 Cracow, Poland

**Keywords:** COVID-19, inherited metabolic disorders, rare autoinflammatory diseases, health care providers

## Abstract

COVID-19 pandemic is an organisational challenge for both healthcare providers and patients. People with rare inherited metabolic disorders (IMD) and rare autoinflammatory diseases (AD) are vulnerable patients whose well-being is deeply connected with regular follow-ups. This study aimed to assess how e one year of coronavirus pandemic has impacted the treatment of patients with IMD and AD in Poland. Surveys were distributed to all healthcare providers that coordinate the treatment of IMD and AD patients. Thirty-two responders (55%) answered the survey. They provide care to 1726 patients with IMD/AD, including 246 patients on dedicated treatment. In 35% of units, the regular appointments were disrupted, primarily because of patient infection. In 18 hospitals, remote visits were implemented, but only 66.6% of patients used this form of consultation. In 14/32 hospitals, administration of the therapy was delayed (median: 17.4 days). Forty-four patients suffered from SARS-COV-2 infection, in majority with mild symptoms. However, four adult patients developed complications, and one died following a SARS-COV-2 infection. Although most hospitals managed to maintain regular visits during the pandemic, more comprehensive implementation of telemedicine and switch to oral therapy or home infusions would be a reasonable solution for the current epidemic situation.

## 1. Introduction

Rare inherited metabolic disorders (IMD) and rare autoinflammatory diseases (AD) are a group of chronic and multisystem disorders with onset from the foetal period to adulthood. IMD is a heterogeneous group of c.a. 700 genetic disorders with a prevalence of around 1 in 800 live births. AD is a group of disorders characterized by recurrent, unprovoked inflammation without the typical features of autoimmune diseases (high titer autoantibodies) and the prevalence ranging from 1/10,000 to <1/1,000,000 live births. The low prevalence of the diseases and the variety of symptoms and disabilities result in high healthcare requirements and require multidisciplinary care. A large-scale survey conducted by EURORDIS in 2017 on 3450 patients with rare diseases in Europe demonstrated that 65% of patients had to frequently visit various health, social and local support services in a short time, and 51% of them found it hard to manage [1].

In 2020 healthcare coordination became even more challenging. In December 2019, several cases of pneumonia caused by novel coronavirus were detected in Wuhan, China [2]. By March 2020, severe acute respiratory syndrome coronavirus 2 (SARS-CoV-2) spread worldwide, and the World Health Organization (WHO) classified the outbreak as a pandemic [3]. The coronavirus disease (COVID-19) placed big pressure on the healthcare system and has changed the organisation of almost every hospital all over the world. Emergency departments and intensive care units were overwhelmed by COVID-19 patients, which caused that even patients with acute disorders like stroke [3] or heart attack [4] had problems getting proper help.

Decreased healthcare availability and fear of SARS-CoV-2 infection caused a series of new challenges for people with rare metabolic diseases. Reports from organizations of rare disease patients worldwide [5,6,7,8,9,10,11,12,13,14], and rare disease healthcare providers [5,15] showed the scale of disturbances: from cancelled appointments, postponed i.v. treatments and hospitalisations to shortage of medical supply and impact on mental health. EURODIS [13] and MetaBERN [5] studies involved Polish patients and health care providers, but the general report about the impact of the pandemic on patients with rare metabolic diseases in Poland is missing.

This study aimed to assess how outpatient clinics and hospitals, which provide care to patients with rare metabolic diseases, functioned and reorganised during one year of the COVID-19 pandemic in Poland and how the pandemic impacted rare metabolic disease patients.

## 2. Materials and Methods

It is a retrospective observational study coordinated by the Coordinating Team for Treatment of Ultra Rare Diseases in Poland. The team consists of specialists responsible for the qualification and monitoring of the treatment of patients with IMD and AD in Poland. The medical procedures in all patients are performed according to disease-specific therapeutic protocols. The study was carried out from 02 March to 30 May 2021 and assessed the first year of the COVID-19 pandemic.

**Figure 1:**
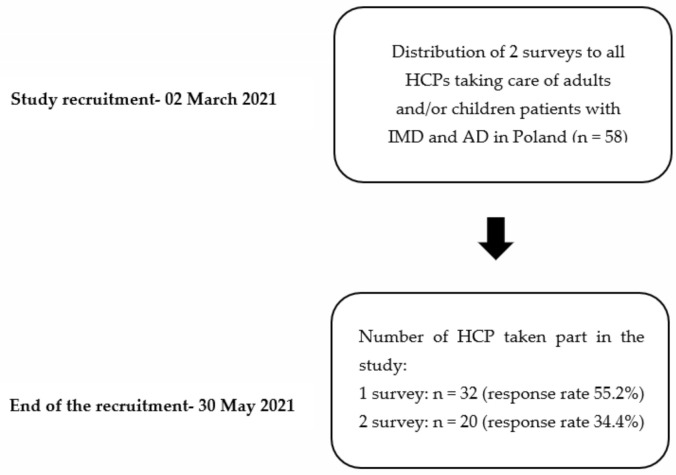
Flowchart of the study (HCP—health care providers; IMD—inherited metabolic disorders; AD—autoinflammatory diseases).

The authors distributed two surveys via email (available as Supplementary Materials) with 30 questions to all 58 health care providers (HCP) taking care of adults or children patients with IMD and AD in Poland. The responders were coordinating physicians responsible for treating the patients (Figure 1).

The multiple-choice and open-ended questions included three major topics: 1. Demographic data on patients, COVID-19 morbidity and its impact on patients health and follow-up (Survey S1). Additionally, the survey included a more detailed questionnaire on patients’ whose therapy is reimbursed in Poland and who are hospitalised regularly (weekly or biweekly) according to a disease-specific therapeutic protocol: Gaucher disease (GD) type I and III, mucopolysaccharidosis (MPS) type I and II, Fabry disease, Pompe disease, tyrosinemia type I, IMDs requiring L-carnitine supplementation, hyperhomocysteinemia, and congenital autoinflammatory syndromes. 2. Organisational changes in health care units, including the implementation of telemedicine (Survey S1). 3. The impact of the pandemic on the diagnosis of new cases / routine admissions to a hospital (Survey S2). 

The study protocol was approved by the Jagiellonian University Bioethical Committee and was in accordance with the Declaration of Helsinki. Informed consent was obtained from all individual participants included in the study.

IBM SPSS (Statistic for Windows, Version 25.0. IBM Corp, Armonk, NY, USA). was used for the data analysis.

## 3. Results

### 3.1. General Information

The response rate of the first survey was 55.2%. The data were collected from 32 HCP, including six strictly paediatric units, 21 centres providing healthcare only for adult patients, and five that follow both paediatric and adult patients. The majority of the clinics followed up 1–4 patients, and only four centres treated more than 50 patients (Table 1). Among all patients, 32.5% were adults, while 67.5% were pediatric patients.

In centers treating both children and adults, the percentage of pediatric patients varied from 69% to 91%. In total, all responders provided treatment to 1726 patients with rare inherited metabolic disorders and rare autoinflammatory diseases (Table 1). The patients received dedicated therapy: enzyme replacement therapy (ERT), substrate-reducing therapy, L-carnitine, anhydrous betaine, nitidinone, or anakinra. The cohort assessed in this study (whose therapy is reimbursed in Poland) included GD type I (40 patients), GD type III (12 patients), MPS I (3 patients), MPS II (18 patients), MPS IV (2 patients), Fabry disease (14 patients), Pompe disease (9 patients), tyrosinemia type I (17 patients), hyperhomocysteinemia (25 patients), diseases requiring L-carnitine supplementation (185 patients) and autoinflammatory diseases (30 patients). Sixty-nine percent of the cohort mentioned above were included in therapeutic programs treatment over some time from March 2020 to May 2021.

The response rate of the second survey was 34.4%. Only 20 HCP answered the questions about the pandemic impact on the diagnosis of new cases of IMD/AD. IMD, inherited metabolic disorders; AD, autoinflammatory diseases.

### 3.2. Health Care Units’ Functioning during the Pandemic

**Figure 2:**
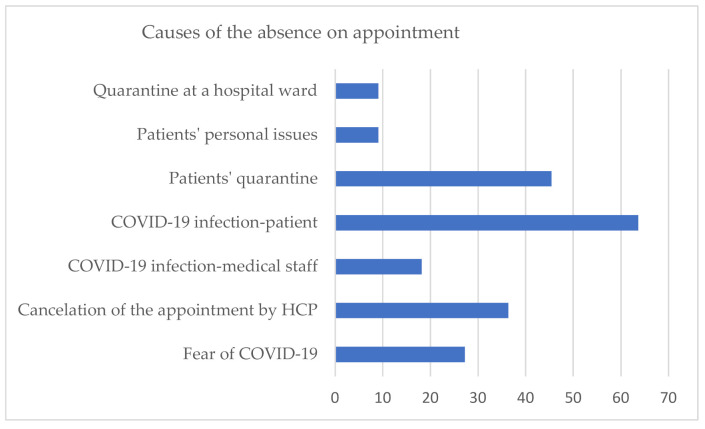
The causes of absence on appointments (HCP—health care providers).

Despite many restrictions and limitations during the COVID-19 pandemic, the majority of HCP (65%) reported that they could continue routine ambulatory visits of patients. Moreover, the continuity of visits was maintained in 7/9 units converted into temporary hospitals dedicated for SARS-COV-2 patients. The causes of postponement or cancellation of appointments in 12/32 cases are shown in Figure 2.

**Figure 3:**
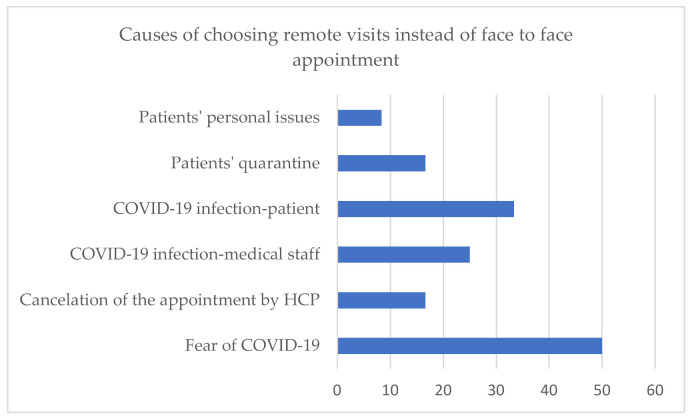
Causes of choosing telemedicine instead of “face-to-face” appointment (HCP—health care providers).

For regular ambulatory visits, 56% (18/32) of HCP implemented remote visits as an alternative, and patients decided to use this form of consultation in 12/19 centres. Five HCP, which follow 1–4 patients each, did not introduce remote visits. The option of a remote visit was introduced in 50% (3/6) paediatric centers, 100% (5/5) units that treat both children and adults, and 52.4% (11/21) centres that follow adults only. The reasons why patients chose remote visits instead of face-to-face appointments are shown in Figure 3.

**Figure 4:**
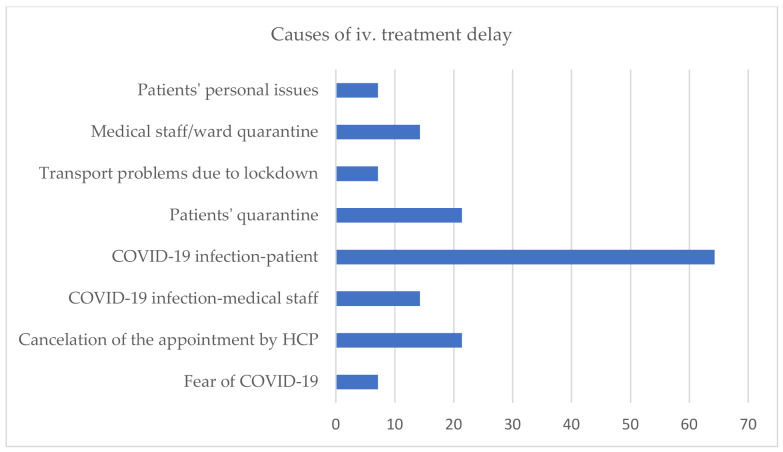
Causes of ERT delay (HCP—health care providers).

In 43.8% (14/32) of HCP, ERT administration was delayed for several reasons (Figure 4). The median time of delay was 17.5 days (7–56 days). In one hospital out of nine, which was converted into COVID-19 unit, there was a treatment delay caused by the cancellation of the visit by hospital authorities (21 days of delay). In another COVID-19 unit, disruptions were caused by fear of COVID-19 and patients’ infection (maximum delay in this unit was 30 days). Other COVID-19 units (78%, 7/9) reported no treatment delay. Importantly, none of the healthcare units reported discontinuation of therapy during the COVID-19 pandemic.

Two clinics reported that they succeeded in switching intravenous to oral therapy in two patients with GD. Also, two hospitals started home delivery of oral drugs for the first time in Poland.

### 3.3. Pandemic Impact on the Diagnosis of New Cases/Routine Admissions to Hospital

Only one hospital (1/20) reported that they experienced a delay in diagnosing new cases of rare diseases due to the pandemic. In the above case, the fear of infection resulted in postponing the planned hospitalisations. In the rest units (19/20), the number of new patients recently diagnosed or transferred from other hospitals was comparable with the time before the pandemic. This number includes all four hospitals with more than 50 rare disease patients. In 10% of healthcare units, fear of COVID-19 infection and patients’ SARS-COV-2 infection resulted in the delay in qualifying patients for the treatment protocol.

Worsening of main disease symptoms, not related to SARS-COV-2 infection, was described in two cases of cystinosis. One patient experienced progression of ocular symptoms because of the postponement of a visit to the ophthalmology outpatient clinic. Another patient had eye complications, and kidney transplantation was delayed in this case.

### 3.4. Patients and SARS-COV-2 Infection

By the end of May 2021, 2.5% of all reported IMD and AD patients suffered from SARS-COV-2 infection confirmed by PCR test, including 25 adults and 19 children (Table 2). In 9 pediatric patients, data about the diagnosis of the main metabolic disease and on the course of infection are not complete. Therefore, these patients are excluded in further analysis. 

Most patients reported fever (62.5%), rhinitis (59%), and general fatigue (56%) as the main symptoms. Four adult patients reported dyspnoea. In two cases it was associated with saturation decrease - one adult patient with Niemann Pick type C (NPC) required hospitalisation in the intensive care unit. Despite ventilator therapy, the patient died due to respiratory failure caused by massive bilateral pneumonia in the course of COVID-19. Additionally, one paediatric patient with very long-chain acyl-CoA dehydrogenase deficiency (VLADD) was hospitalised because of dehydration and metabolic decompensation during SARS-COV-2 infection. Two adult patients needed to be admitted to the hospital due to a thromboembolic event: massive deep vein thrombosis of lower limbs (a woman with Gaucher disease type 1) or ischaemic stroke (a man with Fabry disease type 1) (Table 2). The majority of reported COVID19 (+) patients (20 cases; 60%) received IMD/AD treatment. During SARS-COV-2 infection, nine of them did not have any delay in drug administration, whereas the other 11had the treatment postponed from 7 up to 56 days (median: 17.5 days). Only one hospital reported worsening of the symptoms of the primary metabolic disease due to SARS-COV-2 infection. These were two cases of patients with NPC and one NPB disease. The first patient presented with psychomotor agitation during and after the infection. Two other patients, after a relatively mild course of COVID-19, revealed deterioration of motor abilities (less stable gait, slower speech, noticeably reduced concentration) in the absence of new MRI changes in the CNS.

## 4. Discussion

Patients with rare diseases are a vulnerable group whose everyday functioning and access to healthcare drastically changed during the COVID-19 pandemic. For individuals receiving treatment in hospitals, every one or two weeks, the beginning of lockdown and the rising number of SARS-COV-2 patients caused the fear that their regular treatment would not be continued [16]. According to our data, in almost half of healthcare units (43.7%) that follow IMD and AD, the regular iv. infusions were disrupted. Those data are in line with the Italian study [8] concerning the same profile of patients (lysosomal storage disorders): 49% of patients (out of 102) ERT in hospitals experienced treatment disturbances. In another multicenter study regarding the impact of the COVID-19 pandemic on the diagnosis and management of IMD from a global perspective, the percentage of change of the total number of patients who received specialised treatment (particularly ERT) was 40% [17]. Moreover, a large study conducted by EURORDIS on rare disease patients in Europe revealed that a similar proportion of individuals (49.8% from 6945 patients) were unable to receive therapies such as infusions or chemotherapies [14]. Although the percentage of therapy disruptions was similar, their causes were surprisingly different. In this study, the main reason for treatment delay was a SARS-COV-2 infection, patient quarantine, or cancellation of the appointment by the hospital. Interestingly, fear of COVID-19 infection as a reason for treatment delay was reported only in one hospital (1/32), which was converted into a strictly COVID-19 unit. The rest of the hospitals dedicated to patients with SARS-COV-2 (8/9) did not report treatment delay for that reason, and in all but one, they managed to create separate spaces for rare disease patients to maintain planned infusions without any delay. In similar questionnaires from other countries from the first months of 2020, fear of COVID-19 infection is listed as one of the main causes of therapy disruptions [8,13,18]. These differences may result from the fact that the first wave of the pandemic in Poland was relatively mild compared to other European countries. Therefore, hospitals had sufficient capacity to organise the administration of drugs so that patients felt safe and got used to the new situation until the next waves. 

Many healthcare units outside Poland provided home therapy for people on iv. treatments [5,8,15]. This solution was an excellent way to reduce the risk of treatment discontinuation during a pandemic [8]. Therefore, many HCP encouraged their patients to switch to that form of therapy [5,15]. In Poland, intravenous home therapy is not allowed in the case of rare metabolic diseases, even in the pandemic period. Only two hospitals reported that they started home delivery of oral drugs (tablets) after March 2020. A recent survey performed by the Polish Fabry Disease Collaboration Group [16] showed that 80% of patients with Fabry disease would prefer home infusions of ERT rather than in the hospital, and the majority would not change their preferences after the pandemic. Another way to make therapy more accessible during the pandemic is switching i.v. treatment into oral one as soon as this type of therapy is available, and the patient’s clinical condition allows such a change. Such a possibility exists in the case of Gaucher and Fabry disease, where ERT therapy can be converted to a more convenient, oral substrate reduction/chaperone therapy. By May 2021, such a change in therapy was made in two adult patients with Gaucher disease in two independent hospitals. In both cases, it was done at the patient’s request.

The number of healthcare units that reported disturbances in regular outpatient visits is lower than the number of hospitalisation disruptions (11 vs. 14). It is surprising when considering the fact that therapeutic programmes in Poland have strict organisational and administrative rules, and missed doses may have a serious health impact. On the other hand, patients might experience greater discomfort during hospitalisation than during a visit to the outpatient departments. In the MetaBERN study [5], 54,8% of health care providers of inherited rare metabolic diseases claimed to have a 75–100% of missing/postponed visits. However, there is a visible disproportion between health care units and patients’ responses. In the same MetaBERN study, patient organisations responded that nearly 90% of the visits were postponed. In other similar surveys, the percentage of disrupted appointments based on answers of rare disease patients varied from 53% [11] , through 67% [14] ,71% [9,19] up to 79% [12]. A probable reason for this disproportion is that healthcare providers answered only on behalf of their units, and patients visit disturbances might take place e.g. in GP/other specialist ambulatories. Considering the reasons for appointment disturbances, the most common were: patients SARS-COV-2 infection or quarantine (50%, 36%), cancellation of appointment by health care providers (29%), and fear of COVID-19 (21%). Unlike in other countries [14], fear of COVID-19 was not a crucial factor that caused patients to miss their appointments in Poland, and the survey results about remote visits confirm the results above. Only in 33% (6/18) of the centers allowing for remote visits, the patients chose this form because of fear of COVID-19 infection. Generally, access to telemedicine was provided by 56% of all responding physicians. It is less than in other countries, where the percentage of centres with telehealth options was 90% [5], and about 70% of patients experienced this form of consultations [6,9]. Both healthcare providers and patients claim telemedicine is a useful strategy and should continue in certain cases (e.g. electronic prescriptions) after the pandemic [6,9,13,16].

Patients with IMD, who often suffer from multiorgan dysfunctions, may be at risk of acute or chronic metabolic decompensation, and an infection may trigger even life-threatening episodes. At the beginning of the pandemic, rare disease experts were concerned about the impact of SARS-COV-2 infection on those patients [5]. In this survey, all healthcare providers reported 42 cases of COVID-19 infections of their patients. The coronavirus morbidity in investigated population is much lower than in the general population of Poland in the period from March 2020 to May 2021 (24.7/1000 vs. 76.25/1000) [20]. Those numbers might be underestimated because, in some cases, patients might have limited access to SARS-COV-2 diagnostic tests, even when they developed typical coronavirus symptoms. Moreover, paediatric patients are the majority of the described population, and testing in this age group was uncommon. Additionally, patients who are not receiving i.v./s.c. treatment in hospitals might not always contact their treatment centre in case of infection. 

Almost all patients with infection reported mild, typical COVID-19 symptoms [21]. Those observations are in line with reports from other health care units worldwide [5,9,22,23,24,25]. Only in three cases, the worsening of primary disease symptoms was observed (two patients with NPC and one NPB: psychomotor agitation or deterioration of motor abilities), despite the continuation of symptomatic treatment during infection. 

Coagulation abnormalities leading to thromboembolic complications are observed widely in patients with coronavirus infection [26,27]. One patient with Fabry disease was admitted to the hospital because of ischemic stroke symptoms, and routine tests revealed that he had a SARS-COV-2 infection. People with Fabry disease have a greater risk of developing an ischemic or hemorrhagic stroke [28] due to complex pathophysiology mechanisms leading to endothelial dysfunction and the development of chronic inflammation [29]. Therefore, the association between SARS-COV-2 and stroke is uncertain in this case. Another hospitalised patient was a woman with Gaucher disease type I, who developed massive deep vein thrombosis, probably related to coronavirus infection. 

One patient with NPC revealed rapid respiratory failure caused by massive bilateral pneumonia in the course of COVID-19 and died. The observed worsening of primary disease symptoms and the severe course of coronavirus infection in patients with NPC opposes the hypothesis, claiming that inhibiting NPC1 enables a multistep blockade of viral entry and might be the treatment target for SARS-COV-2 [30,31]. That is a preliminary observation and requires further investigation. Nevertheless, our report draws attention to the group of adult NPC patients as those whose symptoms may worsen in SARS-COV-2 infection.

Interestingly, a large representation of adult patients with AD suffered from COVID-19 (8/30)—all of them with a mild course of the disease. This observation conforms to other studies, suggesting that the anti-inflammatory treatment may ameliorate the symptoms of COVID-19 infection [32,33].

Restrictions during the pandemic had an impact on already diagnosed patients and on diagnosing new ones. In Italy, the number of newly diagnosed patients with rare diseases in the first four months of 2020 was significantly lower than in 2019 and 2018 [15]. Similarly, a significant reduction (76%) of a number of established new IMD diagnoses was reported globally [17]. According to the records from the surveys, in Poland, only one hospital experienced a delay in diagnosing and two reported disturbances in starting i.v. therapy. All four hospitals that follow more than 50 patients reported that the process of diagnosis and qualification for therapy in 2020 was similar to 2019, and the number of diagnosed patients in both years was comparable. However, only 20/32 health care providers answered questions related to that topic. Moreover, 11/20 of those healthcare units followed only one patient and did not perform diagnosis or treatment qualification for years. Therefore, coronavirus pandemic’s real impact in Poland on undiagnosed patients with IMD and AD remains unclear.

The study has several limitations. The survey focuses on physicians’ points of view, highlighting the organisational aspect and general statistics. A special questionnaire dedicated to patients and caregivers of patients with inherited rare metabolic diseases would be crucial. It would help to assess healthcare availability outside main healthcare providers or causes of absence on appointments and, e.g. mental health problems during the pandemic or access to information about COVID-19. Secondly, not all hospitals that provide healthcare to patients with IMD and AD completed the entire survey. However, the response rate of 55.2% of the first survey makes the results of this study representative.

To conclude, although most hospitals managed to maintain the regularity of visits during the pandemic, wider implementation of remote visits and switch to oral therapy or home infusions would be a good solution to improve patients’ health status.

## Figures and Tables

**Table 1:** Patients and healthcare units.

No of Patients			
	Total	On Treatment	SARS-COV-2 (+)
Pediatric	1101	137	19
Adult	625	109	25
Group of IMD patients followed at the centre			
	No of healthcare units		
Only pediatric	6		
Only adult	21		
Both	5		
Number of treated patients in the centre			
	No of healthcare units		
1	12		
2 to 4	11		
5 to 50	5		
50 and more	4		

**Table 2:** Characteristic of patients with COVID-19 infection.

Diagnosis	No of Adults	No of Children	Severity of COVID-19 Symptoms	No of Hospitalised Patients	Cause of Hospitalisation
MPS II	0	6	mild-6/6	0	n/a
MPS IV	1	0	moderate (dyspnea) 1/1	0	n/a
Gaucher disease t. I	4	0	mild-3/4, moderate 1/4 (dyspnea)	1	massive deep vein thrombosis (after SARS-COV-2 infection)
Gaucher disease t. III	0	1	mild 1/1	0	n/a
Fabry disease	3	2	mild 5/5	1 (adult)	ischemic stroke during SARS-COV-2 infection
Pompe disease	2	0	mild 2/2	0	n/a
NPC	4	0	mild 3/4death 1/4	1	respiratory failure caused by massive bilateral pneumonia in the course of COVID-19
NPB	1	0	mild 1/1	0	n/a
Cystinosis	1	0	mild 1/1	0	n/a
VLCADD	0	1	moderate 1/1	1	dehydration and metabolic decompensation
Methylmalonic acidemia	1	0	moderate 1/1 (dyspnea, saturation decrease)	0	n/a
FCAS	3	0	mild 3/3	0	n/a
CAPS	3	0	mild 3/3	0	n/a
Schnitzler syndrome	2	0	mild 2/2	0	n/a

(MPS—mucopolysaccharidosis, NPC—Niemann Pick disease type C, NPB—Niemann Pick disease type B, VLCADD—Very long-chain acyl-CoA dehydrogenase deficiency, FCAS—Familial cold urticaria, CAPS—Cryopyrin associated periodic syndrome).

## Data Availability

The data presented in this study are available upon request from the corresponding author. The data are not publicly available due the specificity of the study, which concerns only Poland. This is a questionnaire survey and there are no standardized and generally available databases on this topic.

## Supplementary Materials

The following are available online at https://www.mdpi.com/article/10.3390/jcm10214862/s1, Survey S1: COVID-19 and rare metabolic diseases -PART I; Survey S2: COVID-19 and rare metabolic diseases -PART II.

## Abbreviations

IMD	rare inherited metabolic disorders
AD	rare autoinflammatory diseases
HCP	health care providers
GD	Gaucher disease
MPS	mucopolysaccharidosis
ERT	enzyme replacement therapy
NPC	Niemann Pick disease type C
NPB	Niemann Pick disease type B
VLCADD	Very long-chain acyl-CoA dehydrogenase deficiency
FCAS	Familial cold urticaria
CAPS	Cryopyrin associated periodic syndrome
