# Supplementary material for: COVID-19 Pandemic and Patients with Rare Inherited Metabolic Disorders and Rare Autoinflammatory Diseases—Organizational Challenges from the Point of View of Healthcare Providers
Source: J Clin Med. 2021 Oct 22;10(21):4862. doi: 10.3390/jcm10214862 (PMC8584872; doi:10.3390/jcm10214862)
Supplement: Supplementary file 1 [file jcm-10-04862-s001.zip › jcm-1393393-Survey S1.pdf]

COVID-19 and rare metabolic diseases -PART I

1. How many patients with a rare metabolic diseases are treated in your Clinic? [open question]

2. How many of those are: pediatric[open question], adult [open question]

3. Please indicate how many patients with a given disease are under the care of your Clinic and how many suffered from SARS-COV-2 infection

	Disease	All patients in the Clinic		Patients after SARS-COV-1 infection	
		pediatric	Adult (>18 y.o.)	dzieci	dorośli >18 lat
1	Gaucher disease t. I				
2	Gaucher disease t. III				
3	MPS I				
4	MPS II				
5	MPS VI				
6	Pompe disease				
7	Fabry disease				
8	Hyperhomocysteinemias				
9	Tyrosinemia				
IMDs requiring L-carnitine supplementation:					
11	3MCC/MCG				
12	GA1				
13	IVA				
14	MMA				
15	PA				
16	LCHAD				
17	VLCAD				
18	MTP, CACT, CPT2				
19	MCAD				
20	MADD				
21	CUD				
22	Autoinflammatory diseases				

4. How many Patients in your Clinic are included into therapeutic programs with reimbused treatment [open question]

5. Has your Clinic been converted into COVID-19 unit during pandemic?

YES NO

6. Did patients regularly visited your Clinic during COVID-19 pandemic (also the metabolic diseases outpatient clinic)?

YES NO

7. If NOT, what was the reason for the absence?

a. Patients' fear of infection in the hospital

b. Cancellation of the appointment by health care providers

c. SARS-COV-2 infection- medical staff

d. SARS-COV-2 infection- patient

e. Quarantine after COVID-19 exposure- patient

f. Other - please specify [open question]

8. Has the telemedicine been launched as part of the patient care?

YES NO

9. Did the Patients use this contact option?

YES NO

10. If YES, what was the reason for choosing this form of visit (you can choose several options).

a. Patients' fear of infection in the hospital

b. Cancellation of the appointment by health care providers

c. SARS-COV-2 infection- medical staff

d. SARS-COV-2 infection- patient

e. Quarantine after COVID-19 exposure- patient

f. Other - please specify [open question]

12. Was there a delay/ disruption in intravenous treatment (f.e ERT) ?

YES NO

If YES, how long? [open question]

13. What was the cause of the interruption / delay?

a. Patients' fear of infection in the hospital

b. Cancellation of the appointment by health care providers

c. Delay in processing of documents

d. SARS-COV-2 infection- medical staff

d. SARS-COV-2 infection- patient

e. Quarantine after COVID-19 exposure- patient

f. Other - please specify [open question]

14. Was worsening of the symptoms of the main disease observed?

YES NO

15. In the case of diseases in which drug programs include oral medications in addition to intravenous infusions - were there any cases of switch;

YES NOT APPLICABLE

If YES, with what effect? [open question]

16. What were the reasons for switching from intravenous to p.o. (you can choose several answer options)

a. Decision of the physician

b. Patient's request

c. A more convenient model of therapy

d. Safety for the patient [open question]

e. Allergic reaction to previously used medication

f. Other - please specify

17. Has any of the patients with rare metabolic diseases/autoinflamatory diseases a Sars COV2 infection?

YES NO

18. What were the symptoms during the infection - please complete an individual questionnaire:

**The symptoms**	**YES**	**NO**
Fever		
Dry cough		
Rhinitis		
Sore throat		
General weakness		
Muscle pain		

Joints pain		
Conjunctivitis		
Headache		
Diarorhea		
Loss of taste or pain		
Skin rush of hands and feet		
Other (please specify)		
**Has any of those symptoms occurred?**		
Shortness of breath or dyspnea		
Saturation decrease		
Chest pain		
Other (please specify)		
**COVID-19 treatment**		
Has the patient been hospitalized?		
What was the COVID-19 treatment? [open question]		
**The treatment of main disease**		
Was the patient treated with intravenous therapy (f.e ERT)?		
Was there any delay/disruption in treatment?<br>If YES, how long? [open question]		
Did you observed the worsening of the symptoms of main disease? Describe the symptoms. [open question]		
