# Supplementary material for: COVID-19 Pandemic and Patients with Rare Inherited Metabolic Disorders and Rare Autoinflammatory Diseases—Organizational Challenges from the Point of View of Healthcare Providers
Source: J Clin Med. 2021 Oct 22;10(21):4862. doi: 10.3390/jcm10214862 (PMC8584872; doi:10.3390/jcm10214862)
Supplement: Supplementary file 1 [file jcm-10-04862-s001.zip › jcm-1393393-Survey S2.pdf]

COVID-19 and rare metabolic diseases -PART II

1. Has COVID pandemic caused delay in diagnostics new patients with rare metabolic diseases.

YES NO

<u>If yes</u>

2. Please mark which rare diseases were diagnosed and how many subjects

	Disease	All patients in the Clinic	
		Pediatric	Adult (>18 y.o.)
1	Gaucher disease t. I		
2	Gaucher disease t. III		
3	MPS I		
4	MPS II		
5	MPS VI		
6	Pompe disease		
7	Fabry disease		
8	Hyperhomocysteinemias		
9	Tyrosinemia		
IMDs requiring L-carnitine supplementation:			
11	3MCC/MCG		
12	GA1		
13	IVA		
14	MMA		
15	PA		
16	LCHAD		
17	VLCAD		
18	MTP, CACT, CPT2		
19	MCAD		
20	MADD		

21	CUD		
22	Autoinflammatory diseases		

3. What was the cause in delay of diagnostics
    a. Fear of infection at a hospital – didn't come for an appointment
    b. Appointment cancelation by management of the facility
    c. Procedural causes – prolonged material flow to the laboratory, extended period of gene marking
    d. Personel illness
    e. Patient's infection
    f. Quarantane due to COV2 exposure
    g. Other – please specify [open question]

4. How many new patients per year are diagnosed at your facility.

	Before COVID-19 pandemic		During COIVD- 19 pandemic	
	Pediatric	Adult (>18 y.o.)	Pediatric	Adult (>18 y.o.)
Newly diagnosed patients				
Transfered patients with diagnosis				

5. Has COVID-19 pandemic delayed inclusion to the treatment of patient with rare metabolic disease

YES NO

<u>If yes</u>

1. Mark which diseases and how many subjects were concerned

	Disease	All patients in the Clinic	
		Pediatric	Adult (>18 y.o.)
1	Gaucher disease t. I		
2	Gaucher disease t. III		
3	MPS I		
4	MPS II		

5	MPS VI		
6	Pompe disease		
7	Fabry disease		
8	Hyperhomocysteinemias		
9	Tyrosinemia		
IMDs requiring L-carnitine supplementation:			
11	3MCC/MCG		
12	GA1		
13	IVA		
14	MMA		
15	PA		
16	LCHAD		
17	VLCAD		
18	MTP, CACT, CPT2		
19	MCAD		
20	MADD		
21	CUD		
22	Autoinflammatory diseases		

6. What was the cause of delay of inclusion to the treatment

   a. Fear of infection at a hospital – didn't come for an appointment
   b. Cancelation of hospitalization by hospital
   c. Procedural causes
      i. prolonged documentation flow,
      ii. extension of gene/biochemic marking
      iii. no posibility of appointment with a specialist
      iv. limited access to imaging and functional examination (MRI, USG, spirometry, etc.)

v. other [open question]

d. Personel infection

e. Patient infection

f. Quarantine after SarsCov 2 exposure

g. Other [open question]

7. How many patients with rare metabolic diseases, not only included in treatment, are cared for by your facility

		Patients	
		Pediatric	Adults >18 y.o.
1	Lysosomal storage diseases (LSD)		
2	Amino and organic-acid related disorders (AOA)		
3	Congenital disorders of glycosylation (CDG)		
4	Carbohydrate, fatty acid oxidation and ketone bodies *disorders* (C-FAO)		
5	Mitochondrial oxidative phosphorylation disorders		
6	Other [which one, open question]		

8. Was there a deterioration of the IMD/AD sympthoms of patients treated in your facility during COVID 19 pandemic.

YES NO

<u>If yes</u>

9. Please mark which diseases and how many patients were concerned

		Patients	
		Pediatric	Adults >18 y.o.
1	Lysosomal storage diseases (LSD)		
2	Amino and organic-acid related disorders (AOA)		
3	Congenital disorders of glycosylation (CDG)		
4	Carbohydrate, fatty acid oxidation and ketone bodies disorders (C-FAO)		
5	Mitochondrial oxidative phosphorylation disorders		
6	Other [which one, open question]		

10. What was the course of exacerbation.

	Diagnosis	The need of hospitalization on the ward dedicated for IMD/AD [YES/NO]	The need of hospitalization on different ward [YES/NO, name of the ward]	Ambulatory treatment [YES/NO]	IMD/AD symptoms similar to those before sars-cov-2 infection [YES/NO]	The worsening of the symptoms of IMD/AD [YES/NO]	The death of the patient [YES/NO]
1							
2							
3							
4							

11. Has the need of hospitalization at other facility than the leading one was due to (check all that apply)
    a. Change of main department to COVID (+) department
    b. Shortage of space at main departement
    c. State of a patient requiring specialized assisstance (ICU, cardiology, etc)
    d. Tranportation of the patiend to a ICU that is the nearest to patient habitation that is outside of the main hospital
    e. Other [open question]

12. What was the cause of exacerbation/progression of IMD/AD

	Diagnosis	Sars-cov-2 infection	Other infection (which one)	Other reason (pleace describe)
1				
2				
3				
4				
